# Cardiovascular Manifestations of Erdheim-Chester's Disease: A Case Series

**DOI:** 10.5935/abc.20180218

**Published:** 2018-12

**Authors:** Isabela Bispo Santos da Silva Costa, André Neder Ramires Abdo, Cristina Salvadori Bittar, Silvia Moulin Ribeiro Fonseca, Aline Sabrina Holanda Teixeira Moraes, Roberto Kalil Filho, Juliana Pereira, Ludhmila Abrahão Hajjar

**Affiliations:** 1 Instituto do Câncer do Estado de São Paulo, São Paulo, SP - Brazil; 2 Instituto do Coração (InCor) - Faculdade de Medicina da Universidade de São Paulo, São Paulo, SP - Brazil

**Keywords:** Erdheim-Chester Disease/diagnosis, Erdheim-Chester Disease/drug therapy, Erdheim-Chester Disease/pathology, Biopsy, Prognosis

## Abstract

Erdheim-Chester Disease is a rare entity, classified as an inflammatory myeloid neoplasm, with an unknown incidence, occurring preferentially in men after 50 years of age. Classically, it has a multisystemic presentation, with the skeletal system being the most frequently affected (90% of the patients), followed by genitourinary involvement in 60% of cases and central nervous system in the pituitary and diabetes insipidus in 25% of the cases. Cardiovascular manifestations are present in more than half of the patients, with aortic infiltration and atrial pseudotumor being the most common forms.

## Introduction

Erdheim-Chester disease (ECD), described by William Chester in 1930, is a histiocytic disorder classified as a member of the L Group, together with Langerhans cell histiocytosis. It is commonly characterized by multifocal osteosclerotic lesions in long bones that shows layers of foamy histiocytes at the histological analysis, accompanied or not by histiocytic infiltration of extra-skeletal tissues.^1^

Its pathophysiology involves the accumulation of xanthoma-like clonal histiocytes (CD68+) in the affected organs. Immune system hyperstimulation by histiocytes causes extensive local and systemic inflammatory reaction^2^ resulting from senescence induction during the oncogenesis process, via hyperactivation of the Ras-Raf-MEK-ERK intracellular signaling pathway. The presence of the BRAF V600E gene mutation is present in up to 2/3 of the patients.^3^

Most patients with ECD are diagnosed between the ages of 40 and 70 years, with a slight predominance of males.^4^ In addition to the long bones, the central nervous system (CNS), cardiovascular system, lung, pancreas, breast, and testicles can also be affected.¹ The cardiovascular involvement occurs in different ways, with periaortic fibrosis being the most common manifestation, with a tomographic finding typically described as a "coated aorta", which is asymptomatic in most patients.^5^ The presence of cardiovascular system impairment is associated with a worse prognosis,^6^ and its identification is of the utmost importance for the adequate management of these patients.

Since the disease has a systemic involvement, it is recommended that all patients be investigated with: 18-Fluorodeoxyglucose positron emission tomography - computed tomography (18-FDG PET-CT), brain magnetic resonance imaging with contrast and detailed examination of the sella turcica and cardiac magnetic resonance (CMR) imaging.^6^ When the CMR is unavailable or contraindicated, a transthoracic echocardiogram (TTE) is performed. Vascular involvement can be assessed through a complementary test to 18-FDG PET-CT with total aorta angiotomography.

### Case reports

Eleven patients with a diagnosis of ECD are followed at our Service. Of these, 4 (36.4%) have cardiovascular disease attributed to the underlying disease; 2 (18.2%) are males, with a mean age of 57 years (38-64 years); 2 (18.2%) patients have cardiovascular manifestation in the form of atrial involvement and aortic involvement; 1 (9.1%) has heart failure with a left ventricular ejection fraction (LVEF) of 40% and 1 (9.1%) has isolated thoracic and abdominal aortic involvement.

### Case 1

A 63-year-old male, diabetic and former smoker patient was referred to the cardiology service one year after the TTE showed an echogenic image suggestive of a mass in the right atrium (RA) measuring 2.5 x 1.3 cm in its largest axis, and increased thickness and density of the atrial septum, suggestive of lipomatous infiltration. Additionally, he had a slight aortic root dilatation, ascending aorta (3.9 cm in diameter) and signs of atherosclerotic plaque in the aortic arch. The complementary CMR showed a solid image in the septal region of the RA, projecting into the mediastinum in the retroaortic position and another image in the region of the RA roof measuring 1.5 x 1.3 cm, adhered to the interatrial septum, with the presence of perfusion and heterogeneous enhancement suggestive of lymphoma.

**Figure 1:**
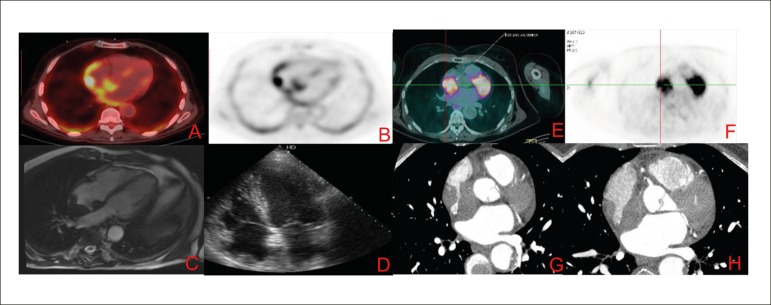
Images A to D refer to case 1 and the images from E to H refer to case 2. Images A and B represent images of 18-FDG PET-CT showing lesion in the right atrium roof. The C image represent CMR image, SSFP cine 4 chambers with hypointense lesion in the right atrium roof. The D image represents a transthoracic echocardiogram image with the same topography. The images E and F represent 18-FDG PET-CT with capturing lesion in the right atrium and G and H images represent contrast computed tomography showing evidence of expansive right atrial.s

The lesion biopsy was carried out; however, the diagnosis was inconclusive. He was referred to the hematology service, where he underwent 18-FDG PET-CT, which identified bone, CNS and skin involvement compatible with ECD. The 18-FDG PET-CT showed a moderate / marked uptake in the RA walls, in topography coincident with CMR alterations, were located on the RA roof (maximum standardized uptake value - SUV_max_: 6.28) and in the interatrial septal region (SUV_max_: 5.65) (Figure 1). Skin biopsy was indicated, of which anatomopathological analysis showed accumulation of xanthomized histiocytes in the dermis, suggestive of xanthelasma, with negative S-100, positive CD68, negative CD1a and positive BRAF V600E staining. The patient underwent initial treatment with interferon, but due to bone disease progression, he is currently undergoing treatment with vemurafenib. In the follow-up 18-FDG PET-CT, RA roof uptake (SUV_max_ = 5.7) was maintained.

### Case 2

This was a 64-year-old female patient, with no prior comorbidities, who was followed by the Hematology team with a diagnosis of ECD, with bone, lymph node and cardiovascular involvement, demonstrated by 18-FDG PET-CT examination. She showed radiotracer hyper-uptake with a heterogeneous pattern in the RA walls (SUV_max_: 5.8) and right ventricle (SUV_max_: 5.8) and discreet pericardial thickening/effusion. The TTE performed in the Cardiology department showed atrial pseudotumor in an echogenic image in the interatrial septum, measuring 2.2 cm x 1.2 cm, suggestive of lipomatous infiltration. The coronary artery angiotomography showed a calcium score (Agatston) of 4, at the 58^th^ percentile of the MESA (Multi-Ethnic Study of Atherosclerosis) study, with no significant coronary luminal reduction. As an additional finding, it showed a soft tissue density expansive lesion in the RA roof related to the interatrial septum and opening into the inferior vena cava. The sinus node artery, the right coronary artery branch, had a partial trajectory through the mass, in addition to atheromatosis in the descending thoracic aorta (Figure 1).

### Case 3

A 38-year-old male patient, with no prior comorbidities, diagnosed with ECD since 2005, identified through lung biopsy with CD68+ histiocyte, negative S-100, started treatment with interferon and prednisone. In 2017, he developed dyspnea at small efforts with NYHA III.

The TTE identified left ventricle (LV) with moderate systolic dysfunction (LVEF of 40%) with diffuse hypokinesia, dilated left chambers, and preserved valvular system. The CMR showed discrete LV dilatation, with an end-diastolic diameter of 6.7 cm and an end-systolic diameter of 5.1 cm, mild diffuse hypokinesia, mild systolic dysfunction (LVEF of 46%) and late enhancement of the junction between the ventricles.

Additional investigations were performed to rule out other etiologies of ventricular dysfunction: serology for Chagas' disease was negative, angiotomography of the coronary arteries with zero calcium score and absence of luminal reduction. Treatment for ventricular dysfunction was started, and the patient showed low tolerance for hypotension and cardiopulmonary rehabilitation was indicated, with an important improvement in dyspnea.

### Case 4

**Figure 2:**
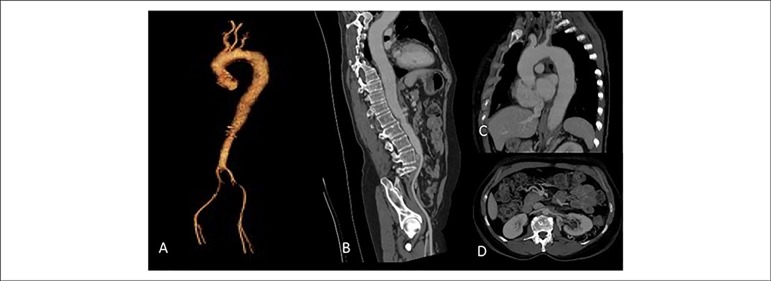
A) Thoracic and abdominal aorta in 3D reconstruction. B) Aorta seen in the sagittal view, showing diffuse thickening of the entire wall with a narrowing area in the infra-renal aorta. C) Thoracic aorta with parietal thickening and luminal reduction in the origin of left subclavian artery. D) Thickening at the origin of the renal arteries, without significant obstruction characterization.

A 63-years-old female patient, a former smoker, with hypothyroidism, arterial hypertension and dyslipidemia, had generalized xanthomatous skin lesions in 2001. In 2004, due to abdominal pain, she underwent a computed tomography (CT) scan of the upper abdomen with contrast, which demonstrated hypoattenuating tissue involving the abdominal aorta and its branches. This promoted a discrete segmental narrowing of some of the vessels characterized by narrowing of the aorta in the emergence region of the renal arteries and the left subclavian artery (Figure 2). Tissue biopsy showed the presence of a pseudotumor, confirming the diagnosis of ECD. The 18-FDG PET-CT showed signs of retroperitoneal fibrosis involving the abdominal aorta immediately above and at the emergence region of the renal arteries. Concomitantly, there was infiltrative tissue surrounding the aortic arch, descending aorta and left common iliac artery. Initially, cardiac involvement had been ruled out by CMR, which had shown normal-sized chambers and preserved systolic function.

## Discussion

DEC is a rare disease, which is difficult to diagnose. The symptoms are varied and not present in all patients. The main complaint is bone pain and there may be fever, night sweats, adynamia, and weight loss, among other symptoms.^7^ These symptoms are not pathognomonic but are useful for assessing treatment response. The interaction of the Cardiology and Hematology services in this scenario allows the adequate diagnosis and management of the cardiovascular system involvement.

The definitive diagnosis is attained through the histological analysis of biopsy samples of affected tissues showing granulomatous infiltration, with CD68 expression, but with negative CD1a staining. Treatment is based on the administration of interferon-( and Vemurafenib, Cladribine and AnA-kinase may be used as the second treatment line, aiming to achieve disease control.^6^

Cardiac involvement in ECD has a worse prognosis and most of the time, it is asymptomatic. Approximately 75% of patients with ECD have some cardiovascular impairment and 60% will be diagnosed with ECD^7^ based on cardiovascular findings, such as in cases 1 and 4 reported above. The cardiologist's knowledge about this disease allows an early diagnosis in these situations.

The most characteristic cardiovascular finding of ECD is aortic involvement,^5^ as seen in case 4, and the most common cardiac lesion is found in the pericardium as pericardial effusion, rarely being associated with cardiac tamponade. The myocardium, endocardium and valvular apparatus may also be involved.

Left ventricular dysfunction, as seen in patient 3, is less frequently observed, but it has also been previously described.^7,8^

Aortic infiltration by ECD is visualized on CT scans as a "coated aorta." This phenomenon results from periaortic infiltration by histiocytes, predominantly in the adventitial layer.^5^ The periaortic fibrosis degree varies from patient to patient, as well as the affected segment. It can occur symmetrically, circumferentially and limited to a specific segment of the aorta or throughout the vessel.

Perivascular infiltration in vessels adjacent to the aorta can also occur in the brachiocephalic trunk, left carotid artery, left subclavian artery, coronary arteries, pulmonary trunk, celiac trunk, superior mesenteric artery, and renal arteries.^7^ The clinical presentation depends on which artery is involved and its degree of stenosis. Cerebral ischemia may occur due to carotid involvement, as identified in case 4, and myocardial infarction due to coronary involvement. Renal artery involvement occurs in approximately 20% of cases^7,8^ and may result in stenosis of these vessels and renovascular hypertension. Treatment is performed through angioplasty and stenting.

Pericardial infiltration can manifest as pericardial thickening with or without fibrosis, and symptoms vary according to the degree of disease severity. Myocardial involvement occurs sequentially to the pericardial involvement and manifests as myocardial hypertrophy, easily diagnosed by the echocardiogram. Thickening can be found in the ventricles, atria, coronary sulci ^7^ and interatrial septum.^9^

Most patients have atrial involvement, often as a pseudotumor, affecting mainly the atrial posterior wall, often projecting into the atrium. Another observed lesion is the infiltration of the right atrioventricular sulcus, where the tissue usually surrounds or infiltrates the right coronary artery.^10^

Haroche et al.,^11^ retrospectively analyzed 37 patients with ECD using CT and CMR: 70% had abnormal cardiac imaging, of which 49% had abnormal infiltration of the right cavities, including 30% with pseudotumor infiltration in the RA, as demonstrated in cases 1 and 2, and 19% with infiltration of the atrioventricular sulcus.

Lipomatous hypertrophy of the interatrial septum (LHIS) is a differential diagnosis that should be considered in some cases, since the TTE often describes the alterations as lipomatous infiltrations. All patients with LHIS show uptake at the 18-FDG PET-CT; however, with smaller mean SUVs (mean of 1.84).^12^ The CMR is an important diagnostic tool in the differentiation of findings, since it can better characterize the tissues. The brown adipose tissue is characterized by hypersignal in T1-weighted images and intermediate signal intensity in T2-weighted images. Specific sequences used to suppress the fat signal, such as the triple inversion-recovery pulse sequence, allow for distinguishing between fatty lesions and other types of tissue.

In another review of 53 patients,^4^ 17% had symptomatic valvular disease, mainly aortic and mitral regurgitation and 3 patients required valve replacement. Technically, surgical valve repair is difficult because of the infiltration of adjacent heart tissue.

ECD is a disease with poor prognosis, with a mean 5-year survival of 68%^5^ and of difficult diagnosis. The scarcity of patients contributes to the lack of knowledge about the disease and the difficulty to develop randomized studies. With this series of cases, we report the largest Brazilian case series to date, focusing on cardiovascular involvement, aiming to contribute to the knowledge of this rare and complex disease.
